# Should the physiotherapy outcomes airway clearance, physical activity and fitness be recorded on the Australian Cystic Fibrosis Data Registry? A consensus approach

**DOI:** 10.1186/s12890-021-01669-2

**Published:** 2021-09-21

**Authors:** Angela Potter, Bhavita Pancholi, Lahni Smith, Carol Maher

**Affiliations:** 1 Physiotherapy Department, Women’s and Children’s Hospital, SA Health, 72 King William Rd, North Adelaide, SA 5006 Australia; 2 Allied Health and Human Performance, University of South Australia, GPO Box 2471, Adelaide, SA 5001 Australia; 3 Alliance for Research in Exercise, Nutrition and Activity (ARENA), University of South Australia, GPO Box 2471, Adelaide, SA 5001 Australia

**Keywords:** Physiotherapy, Airway clearance, Physical activity, Fitness, Health service improvement

## Abstract

**Background:**

Physiotherapy is a cornerstone of cystic fibrosis (CF) management, yet the Australian CF Data Registry (ACFDR) currently does not record physiotherapy-related data. This study aimed to gather opinions from lead Australian CF physiotherapists regarding the importance and feasibility of collecting physiotherapy-related data on the ACFDR.

**Methods:**

A three-round online Delphi survey was conducted to gather expert stakeholder opinion and consensus agreement. Lead physiotherapists from all 23 Australian CF centres were invited to participate. Round one explored the potential benefits, barriers and importance of recording three physiotherapy-related domains on the ACFDR: airway clearance, physical activity and fitness. Subsequent rounds were developed based on the findings from the previous round and sought consensus (80% agreement) for the inclusion of physiotherapy-related data on the ACFDR and for the most appropriate methods of collecting such data.

**Results:**

The response rate was > 80% for all rounds. Participants agreed that collection of airway clearance, physical activity and fitness data on the ACFDR was important and feasible. Findings suggested that airway clearance and physical activity should be collected using self-reported questionnaires, while fitness should be measured using a field-based test.

**Conclusions:**

Australian lead CF physiotherapists believe that collection of airway clearance, physical activity and fitness on the ACFDR is important and feasible. Future work is needed to pilot the data collection procedure to examine its feasibility in real-world clinical settings. This study demonstrates how Delphi methodology can provide a contemporary summary of expert clinicians’ opinion that may underpin nation-wide health service improvement.

**Supplementary Information:**

The online version contains supplementary material available at 10.1186/s12890-021-01669-2.

## Background

Along with medications and disease monitoring, physiotherapy is a cornerstone of cystic fibrosis (CF) management [1]. Traditionally, physiotherapy has focussed on airway clearance therapy, achieved through manual techniques such as percussions, postural drainage and autogenic drainage, which assist individuals with CF to expectorate respiratory secretions and promote lung ventilation [1]. A range of devices may also be used, such as positive expiratory pressure (PEP) and oscillating PEP devices, high frequency chest wall oscilation [2] and, more recently, intrapulmonary percussive ventilation [3]. In recent decades, increasing emphasis has been placed on the role of physical activity and exercise, as an adjunct to, or even substitute for, airway clearance techniques [4].

For the general population, participation in physical activity has wide ranging health benefits and health risk mitigation [5] reflected in national evidence-based physical activity and sedentary behaviour guidelines [6]. For individuals living with cystic fibrosis, exercise has been shown to improve mucociliary clearance (secondary to increased ease of sputum expectoration and improved ventilation and respiratory flow) [7] and maximal aerobic exercise capacity [8]. Participation in physical activity has been associated with a reduction in the decline of pulmonary function over time [9], whilst fitness has a significant positive correlation with quality of life [10] and survival [11]. However, the optimal “dosage” of airway clearance techniques, physical activity and fitness is unclear [8].

Patient data registries offer a valuable source of disease treatment and outcome data which can drive improved patient care [12]. Further benefits of data registries include informing disease care guidelines and monitoring outcomes of interventions and the safety of medications [12]. Accordingly, many jurisdictions worldwide have cystic fibrosis patient registries—the largest being the European CF Society Patient Registry with data from more than 48,000 people with CF [13], and the US CF Foundation Patient Registry (USA) of over 31,000 patients [14]. Canada [15], Brazil [16] and Australia [17] each have CF registries of around 3000–4000 individuals.

Whilst physiotherapy is an integral component of CF management, physiotherapy-related data are currently not consistently recorded on CF patient registries around the world. The CF patient registries of the USA, UK [18] and Canada record airway clearance therapy, whilst the European, Brazilian and Australian CF registries do not. To our knowledge, no CF registries anywhere in the world currently record physical activity or fitness outcomes.

Given the importance of airway clearance techniques, physical activity and fitness in CF management, the notion of recording such data on CF registries appears to hold merit. However, adding new measures to a patient registry is not a simple undertaking. Collection of data on a registry involves a concerted effort—patients must be willing to proffer the data, clinicians must collect it, and someone must enter it onto the database. Such data are only valuable if they are nearly complete; missing data diminishes patient registries' value [19]. These activities must be completed within the health service eco-system, which places competing demands on patients’ and clinicians’ time. This study describes the considerable efforts undertaken to date to explore the potential addition of airway clearance therapy, physical activity and fitness to the Australian CF data registry. In particular, the study aimed to seek lead Australian CF physiotherapists’ perceptions regarding the potential importance and feasibility of collecting airway clearance technique (ACT), physical activity (PA) and fitness data on the ACFDR.

## Methods

### Ethics

This study was approved by the Women's and Children's Hospital, Adelaide, Human Research Ethics Committee (HREC/20/WCHN/64) and was given in principle support from CF Australia (peak body for CF in Australia) and the Monash Data Registry Centre (custodians of the ACFDR) and ACFDR Steering Committee. All methods were carried out in accordance with the relevant guidelines and regulations.

### Study design

A Delphi study approach was used to gather expert opinion and consensus agreement regarding the possibility of the ACFDR collecting physiotherapy data. Delphi approaches involve asking a group of experts their opinions on a particular topic using an iterative structured process. Participants respond to a number of ‘rounds’ of questions or statements, where each subsequent round is based in part on the results of the previous one [20].

### Participant selection and recruitment

The national peak body, Cystic Fibrosis Australia, recognises 22 Australian cystic fibrosis clinics (https://www.cysticfibrosis.org.au/what-we-do/cf-clinics). The lead cystic fibrosis physiotherapist at each clinic was identified by AP either by prior professional contact/knowledge or by phone or email contact to individual clinics. The lead cystic fibrosis physiotherapist for each clinic was targeted based on the rationale that they were the most appropriate physiotherapy representative for their clinic and would have a high level of knowledge and experience regarding physiotherapy data collection. One cystic fibrosis clinic had separate adult and paediatric lead physiotherapists (n = 23 lead physiotherapists).

All 23 lead cystic fibrosis physiotherapists across Australia were contacted via email to introduce the project and its aims in July 2020. Physiotherapists were subsequently emailed formal information regarding project design, time frames, time commitments and consent process. Informed consent was obtained from all the participants’ in the ethics approval section*.*

### Delphi surveys

Three survey rounds were undertaken from July to Sept 2020. Survey 1 was informed by a scoping literature review, and findings from the previous rounds informed surveys 2 and 3. The survey was administered online, using Survey Monkey (www.surveymonkey.com). Each survey round was open for 10 days, with a reminder email sent at day 7. Summary reports were emailed to participants after each round.

#### Round 1

The Round 1 Delphi survey (Additional file 1: Supplementary file 1) comprised 13 items. It collected basic professional information, asking participants to confirm their position as lead cystic fibrosis physiotherapist at an Australian cystic fibrosis centre, identify which client cohort they worked with (paediatric and/or adult) and documenting the number of years of experience working in cystic fibrosis (3 items). Participants were then invited to share their opinions regarding the potential benefits of, and barriers to, recording physiotherapy-related information on the ACFDR, using 6 open-ended items. Physiotherapy-related outcomes were presented in three domains: airway clearance, physical activity and fitness. Participants were then asked to rate the perceived importance of recording ACT, PA and fitness outcomes of the ACFDR using 4-point Likert scales (strongly disagree, somewhat disagree, somewhat agree, strongly agree) (3 items). The survey concluded with an open-ended item for further comments. A priori, progression to Round 2 was deemed appropriate if the majority (> 50%) of respondents agreed that recording physiotherapy-related information was important *(somewhat agree, strongly agree)*.

#### Round 2

The Round 2 Delphi survey (Additional file 1: Supplementary file 2) was developed based on the findings from Round 1. The preamble for the Round 2 survey included a summary report of Round 1 findings including response rate, participant information and participants’ opinions on potential benefits and barriers to collecting physiotherapy data on the ACFDR. In total, the Round 2 survey comprised 17 items. Participants were asked to rate the importance of and comment on the three physiotherapy-related domains for potential inclusion onto the ACFDR—airway clearance (individual techniques used and frequency/compliance), physical activity and fitness. Importance was rated on a 9-point Likert scale, ranging from “not at all important” (1) to “very important” (9). Consensus was defined a priori as ≥ 80% of respondents rating an item as important (≥ 7 on the 9-point scale). There is no universally accepted threshold for defining consensus in Delphi surveys [21]. We chose 80% on the rationale that we would need strong majority support to consider introducing physiotherapy measures to the ACFDR, given that it involves practice change and additional burden for clinicians and patients. The Round 2 survey also started to explore participants' views on how the three domains might be best captured on the ACFDR (further explored in Round 3). For ACTs, participants were asked their opinion on which specific ACTs they believe should be listed on the ACFDR using a yes/no response for common ACTs, and free-text item for nominating additional ACTs for consideration. Consensus for ACT inclusion was deemed to have been reached if ≥ 80% of participants responded affirmatively. Participants were invited to rate the importance of recording ACT frequency/compliance on the 9-point Likert scale and suggest how frequency/compliance might be measured (free text). Next, participants were invited to suggest preferences for measuring PA (wearables *vs* self-report) and comment on the feasibility of collecting PA data (free text). Similarly, participants were asked for preferences for collecting fitness data (laboratory fitness tests *vs* field fitness tests) and comment on the feasibility of collecting fitness data (free text). Finally, participants were asked to give their opinion on the age at which it might be appropriate to start data collecting PA and fitness outcomes.

#### Round 3

The Round 3 Delphi survey (Additional file 1: Supplementary file 3) was developed based on the findings from Round 2. The preamble for the Round 3 survey included a summary report of Round 2 findings including response rate, items that had reached consensus, methods of measuring compliance with airway clearance, tools for measuring physical activity and fitness and patient age at which to commence data collection.

Lead physiotherapists, including those who did not respond to Round 2, were emailed a report which included a summary of themed participant comments and participant response histographs with documentation of their rating for each item.

Participants were asked to re-rate items that did not reach consensus in Round 2 and rate new items developed from participants' comments in Round 2. Items were designed to capture participants’ opinions regarding (1) whether each of the additional airway clearance techniques identified in Round 2 should be listed on the ACFDR (yes/no responses); (2) the importance of measuring frequency/compliance of airway clearances (9-point Likert scale); (3) the feasibility of collecting physical activity data using either self-reported instruments or wearable activity trackers (each scored on a 9-point Likert scale ranging from “not all feasible” (1) to “very feasible” (9)); (4) the importance of capturing PA data on the ACFDR (9-point Likert scale); (5) the feasibility of measuring fitness using either field tests or laboratory-based tests (each scored on a 9-point Likert scale); (6) the importance of capturing fitness data on the ACFDR (9-point Likert scale) and (7) the most appropriate age to commence physical activity and fitness data collection. Participants were invited to record their interest in being involved in a future feasibility study.

### Data analysis

Response rate was calculated as the percentage of participants who completed the survey from all potential participants contacted. Participants’ demographic information (patient population and years of experience) was reported descriptively (using percentages for categorical data, and means, standard deviations and ranges for continuous data (or medians, interquartile ranges and ranges when data were skewed)).

In round 1, participants’ interest in collecting ACT, physical activity and fitness was examined on a 4-point Likert scale, with response categories 3 and 4 collapsed into a single “agree” category. In rounds 2 and 3, 9-point Likert scales were used to examine “importance” and “feasibility”. For analysis, response categories 1, 2 and 3 were collapsed to create a category “not important/feasible”, responses 4, 5 and 6, collapsed to “neutral” and responses 7, 8 and 9 collapsed to “important/feasible”. In all rounds, open-ended responses were analysed in naturally-emerging themes by two reviewers (BP and LS), with disagreements resolved by AP and CM.

## Results

All 23 lead cystic fibrosis physiotherapists were invited to each of the three Delphi rounds.

### Round 1

The response rate for Round 1 was 91% (n = 21/23). Over half the respondents (57%; n = 13) worked in adult-only CF centres, around one third (38%; n = 9) worked in paediatric centres and 5% (n = 1) worked in a centre serving both adult and paediatric patients. On average, participants had been specialising in cystic fibrosis physiotherapy for a mean of 13.3 years (SD 6.1, range 4–25).

Participants were asked their opinions regarding the potential benefits and disadvantages/barriers to collecting information on the ACFDR concerning airway clearance, physical activity and fitness (Table 1). In general, the benefits and barriers raised by participants were similar for each of the three domains. For example, commonly cited benefits were the ability to benchmark/compare centres, analyse outcomes of different physiotherapy interventions and review trends in current physiotherapy practice. Disadvantages/barriers most commonly related to the perceived extra workload for clinicians associated with data collection and data entry and the variability/validity/reliability of outcome measures.

**Table 1:** Potential benefits and disadvantages/barriers to collection of information on the ACFDR

Domain	Potential benefits	Potential disadvantages/barriers
Airway clearance	Would allow for comparison and bench-marking between centres in Australia and overseas (16) Would facilitate analysis of outcomes of different airway clearance techniques (7) Would assist identification of trends over time in chest physiotherapy practice (6) Would allow for centralisation of data collection (6) Would be useful for research purposes (4)	An increase in workload/time required (12) A need to consider/maximise the validity and reliability of outcomes within and across centres (9) A risk of data incompleteness (8) Determining who would be responsible for collecting and entering data at each centre (e.g. physiotherapist vs admin staff) (4)
PA/exercise outcomes	Would allow for comparison and benchmarking between centres in Australia (10) Would assist the identification of trends in physical activity over time (5) May be useful for research purposes (for example, understanding the role of physical activity as an adjunct/substitute for chest physiotherapy) (4) Would allow for tracking of patients' progress (3) Would allow patient and healthcare providers access to data (3)	An increase in workload and time required (14) A risk of variation in validity and reliability of outcomes/regimes across centres/patients (14) A risk of data incompleteness due to the burden of collecting data across Australia (4) The specific environmental space and equipment requirements (4) A risk of increasing patient time and treatment burden (1)
Fitness outcomes	Comparison and benchmarking between centres (9) Understanding of relationships between fitness and disease mortality/morbidity rate (5) Tracking of patients' progress (4)	An increase in workload and time (11) The large range of tests and the need to consider type, timing, reliability and validity when selecting the most appropriate test(s) (8) A risk of data incompleteness due to the burden of collecting data across Australia (7) A risk of variation in validity and reliability of outcomes/regimes across centres/patients (4) The lack of relevance and clinical value of some fitness parameters (e.g. flexibility) (2) The environmental space required to conduct a fitness test (1)

Number of respondents in parentheses

Participants were asked to rate their agreement/disagreement with the value of collecting information regarding airway clearance, physical activity/exercise and fitness on the ACFDR (Table 2). The majority of participants who responded to this section of the survey (n = 18) agreed that collection of data on airway clearance (89%; n = 16), physical activity (72%; n = 13) and fitness (83%; n = 15) outcomes would be valuable, exceeding the a priori 50% agreement threshold required for progression to Round 2.

**Table 2:** Participants' ratings of the value of collecting data on the ACFDR (n = 18)

Statement	Strongly disagree (n, %)	Somewhat disagree (n, %)	Somewhat agree (n, %)	Strongly agree (n, %)
It would be valuable to collect airway clearance outcomes of the ACFDR	2	0	4	12
	11%	0%	22%	67%
It would be valuable to collect physical activity/exercise outcomes on the ACFDR	1	4	3	10
	6%	22%	17%	56%
It would be valuable to collect fitness outcomes on the ACFDR	2	1	7	8
	11%	6%	39%	44%

### Round 2

Nineteen participants (83%) responded to the Round 2 Delphi survey.

Participants rated the importance of recording airway clearance, physical activity and fitness data on the ACFDR on the 9-point Likert scale (Table 3). Consensus was reached for the inclusion of airway clearance on the ACFDR (84% agreement). Consensus was approached, but not reached for inclusion of frequency/compliance of airway clearance (63%; n = 12), physical activity (68%; n = 13) or fitness (79%; n = 15).

**Table 3:** Round 2 responses (n = 19)-importance of recording items on the ACFDR

Item	Percentage of participants scoring the item ≤ 3 on the 9 point scale (“not important”) (n, %)	Percentage of participants scoring the item 4–6 on the 9 point scale (“neutral”) (n, %)	Percentage of participants scoring the item ≥ 7 on the 9 point scale (“important”) (n, %)
How important do you believe it is to collect airway clearance techniques on the ACFDR?	1	2	16
	5%	11%	84%
In your opinion, how important is capturing a frequency/compliance item with airway clearance techniques?	2	5	12
	11%	26%	63%
How important do you believe it is to collect physical activity outcomes on the ACFDR?	0	6	13
	0%	32%	68%
How important do you believe it is to collect fitness outcomes on the ACFDR?	0	4	15
	0%	21%	79%

All proposed airway clearance techniques (active cycle of breathing, autogenic drainage, exercise, high frequency chest wall oscillation, oscillatory positive expiratory pressure, positive expiratory pressure, and postural drainage with percussion) reached consensus threshold for inclusion on the ACFDR (range 84–100% agreement).

Participants also suggested the following additional airway clearance technique options/wording for inclusion on the ACFDR: (1) postural drainage in combination with percussions and vibrations (2) combined treatments, e.g. nebulisers pre/during airway clearance, hypertonic saline (3) further specification of devices, e.g. Aerobika vs Flutter, Hill ROM vs Afflo vests (4) non-invasive ventilation/biphasic positive airway pressure (biPAP) and (5) intrapulmonary percussive ventilation (IPV).

Of the 19 respondents who offered their opinions on measuring the frequency/compliance with airway clearance, 53% (n = 10) recorded concerns about validity, reliability, standardization or accuracy of self-reporting; 42% (n = 8) participants suggested that the number and frequency of treatment completed (sessions/day, time/session) could be measured; and 21% (n = 4) participants suggested recording of the recommended treatment vs actual performance via self-report.

With regard to the proposed methods of measuring physical activity annually, approximately half (53%; n = 10) of participants preferred the use of wearables, while the other half (47%; n = 9) preferred self-report. When asked to comment on the feasibility of measuring physical activity using wearables, numerous barriers were raised, such as concerns about poor validity, likelihood of missing data, problems with accessibility to equipment, technical difficulties, burdensome administration and extra financial costs associated with wearable devices. For self-reported physical activity, the main barriers were lack of validity and accuracy, while the main enabler was their ease of use. Some participants suggested recording both wearable-measured and self-reported PA on the ACFDR. Suggested PA wearables included research-grade accelerometers (e.g. actigraphs (ActiGraph Corporation, Pensacola, Florida), consumer-level wearables (e.g. Fitbits (Fitbit, San Francisco, California)) and smartphone apps. Suggested self-reported PA measurement tools included the IPAQ-S (International Physical Activity Questionnaire short form) [22] and HAES (Habitual Activity Estimation Scale) [23].

With regard to possible approaches for measuring fitness annually, approximately half (47%; n = 9) of participants preferred laboratory-based fitness tests, while the other half (53%; n = 10) preferred field-based tests. When asked to comment on the feasibility of measuring fitness with laboratory tests, numerous barriers were raised: time burden for patients and staff, cost, impracticality and lack of access to the required equipment. For feasibility of field-based fitness tests, barriers such as specificity, ceiling effect, difficulty of finding a test suitable for both adults and paediatric patients, space, time burden for patients and staff and “variability” (presumably relating to questionable inter-rater reliability) were the main themes.

Suggested options for laboratory-based fitness tests included cardiopulmonary exercise testing (CPET) as the gold standard test, Bruce protocol treadmill test, and "ramping bike protocol" (specific protocol not named). Suggested field-based tests included the six-minute walk test, modified shuttle walk test, step tests, modified shuttle test (MST25).

Other comments included the need to monitor SpO2 and heart rate during fitness testing; consideration of the severity of lung disease; usefulness of the data in terms of impact on patient care and directing meaningful change in practice; consideration of recording both laboratory and field testing; that field tests may be undertaken via telehealth; and that measuring fitness is now of greater importance in the era of cystic fibrosis transmembrane regulator modulator therapy.

Participants were asked their opinion on the most appropriate age for starting to collect PA and fitness data: the mean recommended ages were 9.3 years (SD 4.1, range 2–20 years) and 10.1 years (SD 3.2, range 6–20), respectively.

### Round 3

Nineteen participants (out of 23, 83%) responded to the Round 3 Delphi survey.

All proposed airway clearance techniques (combined treatments, specific devices and non-invasive ventilation) reached consensus threshold for inclusion on the ACFDR (range 90–100% agreement).

Participants re-rated the importance of recording physical activity and fitness data on the ACFDR on a 9-point Likert scale and rated the feasibility of recording these domains (Table 4). Consensus was reached for the importance of including physical activity and fitness data on the ACFDR (89% and 95% respectively). The importance of recording frequency/compliance of airway clearance narrowly missed consensus (79% agreement). Concerning the feasibility of collecting physical activity information, self-reported methods were considered feasible whilst wearable devices were not (95% vs 37% agreement respectively). Collecting fitness data using field-based tests was more feasible than laboratory-based tests (74% vs 42% agreement, respectively).

**Table 4:** Round 3 responses (n = 19)-importance and feasibility of recording items on the ACFDR

Item	Percentage of participants scoring the item ≤ 3 on the 9 point scale (“not important/not feasible”) (n, %)	Percentage of participants scoring the item 4–6 on the 9 point scale (“neutral”) (n, %)	Percentage of participants scoring the item ≥ 7 on the 9 point scale (“important/feasible”) (n, %)
How important is capturing a frequency/compliance item with airway clearance techniques?	1	3	15
	5%	16%	79%
How important is capturing physical activity data on the ACFDR?	0	2	17
	0%	11%	89%
How important is capturing fitness data on the ACFDR?	0	1	18
	0%	5%	95%
How feasible would it be for your centre to gather annual physical activity data using wearable devices?	5	7	7
	26%	37%	37%
How feasible would it be for your centre to gather annual physical activity data using a self-report method?	0	1	18
	0%	5%	95%
How feasible would it be for your centre to gather fitness data using laboratory-based fitness tests?	7	4	8
	37%	21%	42%
How feasible would it be for your centre to gather fitness data using field-based fitness tests?	1	4	14
	5%	21%	74%

The majority (63%; n = 12) of participants agreed that the age for collecting physical activity and fitness data should be the same. The most common (58%; n = 11) response was that 10 years of age was the most appropriate age for commencing collection of these data.

## Discussion

This study gathered Australian cystic fibrosis specialist physiotherapists’ perceptions regarding the benefits, barriers, importance and feasibility of collecting physiotherapy data on the Australian Cystic Fibrosis Data Registry. Participants stated that collecting physiotherapy data on the ACFDR would facilitate benchmarking between centres, building an understanding of the outcomes of different physiotherapy interventions and allow identification of trends in current physiotherapy practice. Key barriers to the collection of such data were the perceived extra workload for clinicians associated with data collection and data entry and the variability/validity/reliability of outcome measures. Despite these cited barriers, the Delphi process reached consensus for the collection of data regarding airway clearance therapy, physical activity and fitness. In terms of measurement approaches, participants agreed that it was feasible to measure airway clearance therapy using a purpose-designed question which listed leading airway clearance techniques. Participants agreed that physical activity should be measured using a validated physical activity questionnaire and that fitness should be measured using a standardised field-based test.

Participants’ enthusiasm for the routine collection of physiotherapy-related items was evident from the study’s outset. All Delphi rounds achieved a strong participation rate, and many physiotherapists voiced their support for the initiative, typified by comments such as “*[This is] a great undertaking, I think, generally speaking, it would be an excellent addition to the register.” “It’s a shame it has taken so long for it to be considered given physio[therapy’s] heavy involvement with CF”.* Key benefits to collecting physiotherapy data on the ACFDR were in line with widely recognised benefits of patient registries, such as recording the effectiveness of interventions in real-world settings; providing benchmarked reports to key stakeholders; and measuring and improving quality of care [12].

By the end of the third Delphi round, a consensus was reached for all three domains: airway clearance techniques, physical activity and fitness, though the consensus was reached more swiftly for some domains than others. Collection of airway clearance treatment reached immediate consensus in round 2, with the remaining round focused on capturing all of the key airway clearance technique categories. It was not surprising that airway clearance techniques reached consensus quickly, considering that airway clearance is still a mainstay treatment for cystic fibrosis in Australia [1], and that such data can be gathered readily using a simple purpose-designed item. CF patient registries in the USA, Canada and UK similarly collect data on airway clearance techniques.

Support for the collection of physical activity and fitness data was more mixed in the early Delphi stages. However, the free-text comments suggested that those initially not supportive of measuring these domains were hesitant due to feasibility concerns (i.e. that Australia-wide collection of data using criterion measurement approaches may be impractical, jeopardising data completeness) or concerns regarding the lesser validity of non-criterion measurement approaches. Data incompleteness is an important issue, and a recognised issue with patient data registries [24]. Selecting a measurement approach for clinical settings can require a trade-off between measurement properties (e.g. consideration of the measurement approachs’ validity) and practical considerations (e.g. the tests’ time and cost burden for participants and clinicians). The Delphi participants correctly understood that self-reported physical activity measurement approaches and field-based fitness tests have inferior validity to their criterion counterparts. However, in some cases, it appears they over-estimated these validity limitations. For example, while self-reported physical activity tools have lower validity than accelerometer approaches, they still typically have moderate validity [25] and are recognised as valuable in cases where the more rigorous measurement approaches are time- or cost-prohibited [25, 26]. Similarly, field-based fitness tests can have moderate-to-high validity [27], provided they are administered according to standardised procedures, and are carefully selected for the target population, to avoid floor or ceiling effects [28]. Once these issues were probed in latter Delphi rounds, a consensus for measuring physical activity and fitness was achieved.

A strength of this study was its high participation rate. The study also emphasised the identification of barriers and other practical considerations, which will help ensure the study’s findings are clinically appropriate. A further strength was that the peak body for cystic fibrosis in Australia and the ACFDR Steering Committee endorsed the study. Support from these entities will be crucial enablers should physiotherapy data be added to the ACFDR in future.

The study’s sample size may appear somewhat small; though it was appropriate considering there are only 22 cystic fibrosis centres throughout Australia. The study’s focus solely on cystic fibrosis physiotherapists, and not other stakeholders, may also be considered a limitation. However, establishing the physiotherapists’ position on the potential collection of physiotherapy data on the patient registry is a key first step, given that this group will play pivotal roles in data collection and data entry should this initiative proceed. Now that support from this group has been established, it will be essential to involve other stakeholders in this research project moving forward. Finally, the Delphi study concluded with identifying key characteristics of physical activity and fitness measurement tools. Further work will be needed to identify the precise measurement tools that best meet these needs.

### Implications

This study found there is in-principle support from specialist CF physiotherapists in Australia to collect airway clearance techniques, physical activity and fitness data annually for entry into the ACFDR. Following on from this study, a literature search will be required to identify candidate instruments for collecting self-reported physical activity and field-based fitness. Ideally, such tools will be suitable for both adults and children with CF, have established reliability and validity, and be practical to administer in a standard clinical setting.

While the study revealed strong notional support for collection of these domains, in reality, there will be ongoing barriers, particularly related to clinicians’ time to collect the data and enter it into the ACFDR. Once seemingly appropriate tools for collect airway clearance techniques, physical activity and fitness data have been identified, the feasibility of their administration within the clinical setting will need to be scrutinised. The most appropriate clinical context for data collection will need careful consideration to ensure data are collected outside periods of acute exacerbations, and are collected in a manner that minimises additional patient and clinician burden. Ideally, data collection would dovetail with existing service occasions to avoid the need for an additional hospital visit and occasion of service. Thorough feasibility testing will be needed, taking into account the time commitment and perspectives of patients, families, physiotherapists and the broader multi-disciplinary cystic fibrosis team, all of whom will be impacted by the collection of these data. Feasibility should be examined across multiple cystic fibrosis clinics, given that the way services are delivered is likely to vary between clinics.

Importantly, this study provides an example of a clinically-derived question, which has grown into a nation-wide health service improvement activity, undertaken within an implementation science framework. In future, we envisage this program of research may lead to the collection of physiotherapy-related outcomes on an annual basis for all patients with CF throughout Australia. If it goes ahead, to our knowledge, it is the first initiative to collect physical activity and fitness data with CF patients on a national scale anywhere in the world. Collecting such longitudinal data in a large patient cohort will provide invaluable opportunities to understand better the role of airway clearance techniques, physical activity and fitness in the long term management and health outcomes of patients with CF across the lifespan.

In conclusion, this study used a Delphi approach to generate consensus from specialist CF physiotherapists across Australia regarding the potential collection of airway clearance technique, physical activity and fitness data on the Australian CF Data Registry. Results suggested there is strong support amongst physiotherapists to collect these data provided the airway clearance technique and physical activity data are collected using self-reported instruments, and fitness is measured using a valid field test. The routine annual collection of these data will help reveal the role of airway clearance techniques, physical activity and fitness in cystic fibrosis management and outcomes. Future research examining the feasibility of data collection and data entry is now required.

## Supplementary Information

**Additional file 1**: The Delphi surveys.

## Data Availability

The datasets used and/or analysed during the current study are available from the corresponding author on reasonable request.

## Abbreviations

CF: Cystic fibrosis
ACFDR: Australian Cystic Fibrosis Data Registry
ACT: Airway clearance technique
PA: Physical activity
biPAP: Biphasic positive airway pressure
IPV: Intrapulmonary percussive ventilation
IPAQ-S: International Physical Activity Questionnaire short form
HAES: Habitual Activity Estimation Scale
CPET: Cardiopulmonary exercise testing
