# Supplementary material for: Should the physiotherapy outcomes airway clearance, physical activity and fitness be recorded on the Australian Cystic Fibrosis Data Registry? A consensus approach
Source: BMC Pulm Med. 2021 Sep 21;21:298. doi: 10.1186/s12890-021-01669-2 (PMC8456641; doi:10.1186/s12890-021-01669-2)
Supplement: Supplementary file 1 — Additional file 1: The Delphi surveys. [file 12890_2021_1669_MOESM1_ESM.docx]

SUPPLEMENTARY FILE 1

ROUND 1 DELPHI

Item 1. Are you the Lead CF Physiotherapist at an Australian tertiary CF centre? (yes; no; no, but currently filling in for the lead physiotherapist)

Item 2. What client cohort do you work with? (paediatric; adult; paediatric and adult)

Item 3. How many years have you worked as a CF Physiotherapist?

Item 4. What would be the benefits of the ACFDR collecting physiotherapy outcomes? (free text)

Item 5. What would be the benefits of the ACFDR collecting physical activity/exercise outcomes?

Item 6. What would be the benefits of the ACFDR collecting fitness outcomes?

Item 7. What would be the disadvantages/barriers to the ACFDR collecting physiotherapy outcomes?

Item 8. What would be the disadvantages/barriers to the ACFDR collecting physical activity/exercise outcomes?

Item 9. What would be the disadvantages/barriers to the ACFDR collecting fitness outcomes?

Items 10-12. Please rate how strongly you agree or disagree with the following statements (4 point scale):

It would be valuable to collect physiotherapy outcomes on the ACFDR

It would be valuable to collect physical activity/exercise outcomes on the ACFDR

It would be valuable to collect fitness outcomes on the ACFDR

Item 13. Please feel free to share any other thoughts and comments

SUPPLEMENTARY FILE 2

ROUND 2 DELPHI

Item 1. How important do you believe it is to collect airway clearance techniques on the ACFDR? (9 point scale)

Item 2. Which airway clearance techniques should be listed, in an item intended to capture airway clearance techniques? (yes/no). Active cycle of breathing technique; autogenic drainage; exercise; high frequency chest wall oscillation; oscillating positive expiratory pressure; positive expiratory pressure; postural drainage and percussion.

Item 3. Please comment on the wording/terminology of the airway clearance techniques listed.

Item 4. In your opinion, how important is capturing a frequency/compliance item with airway clearance techniques? (9 point scale)

Item 5. Please comment on which aspects of frequency/compliance should be measured, and how this might be done.

Item 6. Feel free to share any further thoughts in the comment box below on collecting different airway clearance techniques outcomes on the ACFDR.

Item 7. How important do you believe it is to collect physical activity outcomes on the ACFDR? (9 point scale).

Item 8. Please select your preferred approach, wearable or self-report, bearing in mind that this data would be captured annually for all CF patients. (wearables/self-report).

Item 9. Please comment on the feasibility of measuring physical activity with wearables or self-report. You may name a device or self-report instrument that you have experience with if you think it may be suitable for inclusion in the ACFDR.

Item 10. At what age do you believe it is appropriate to start collecting physical activity data from patients?

Item 11. Feel free to share your thoughts in the comment box below on collecting physical activity outcomes on the ACFDR.

Item 12. How important do you believe it is to collect fitness outcomes on the ACFDR? (9 point scale).

Item 13. Please select your preferred approach laboratory fitness tests or field fitness tests, bearing in mind that this data would be captured annually for all CF patients. (9 point scale).

Item 14. Please comment on the feasibility of measuring fitness with laboratory fitness tests or field fitness tests. You may name a laboratory fitness test or field fitness test that you have experience with if you think it may be suitable for inclusion in the ACFDR.

Item 15. At what age do you believe it is appropriate to start collecting fitness data on patients?

Item 16. Feel free to share your thoughts in the comment box below on collecting fitness outcomes on the ACFDR.

Item 17. Finally, please feel free to use the comment box below to share any other thoughts and comments.

SUPPLEMENTARY FILE 3

ROUND 3 DELPHI

Item 1. Please indicate which additional airway clearance techniques you believe should be included on the ACFDR. (combined treatments, further specific devices, non-invasive ventilation).

Item 2. Please re-rate how important you believe capturing a frequency/compliance item with airway clearance techniques is? (9 point scale)

Item 3. Briefly justify your score in the text box below

Item 4. If a validated, reliable and appropriate wearable was suggested, how feasible would it be for your centre to gather physical activity data using wearables? This would include sourcing and storing the wearables, distributing the wearables to patients annually and collecting them back, and downloading the data. (9 point scale).

Item 5. If a validated, reliable and age appropriate physical activity self-report survey was suggested, that could be distributed annually to patients and their caregivers, and be completed by them in 5-10 minutes, how feasible would it be for your centre to gather physical activity data using the self-report method?

Item 6. Please re-rate how important you believe capturing physical activity data on the ACFDR is? (9 point scale)

Item 7. Briefly justify you score in the text below.

Item 8. If a validated, reliable and age-appropriate laboratory-based test was suggested, how feasible would it be for your centre to gather fitness using laboratory-based fitness tests? (9 point scale).

Item 9. If a validated, reliable and age-appropriate field-based test was suggested, how feasible would it be for your centre to gather fitness data using the field-based test? (9 point scale).

Item 10. Please re-rate how important you believe capturing fitness data on the ACFDR is? (9 point scale).

Item 11. Briefly justify you score in the text below.

Item 12. Do you believe the data collection starting age should be the same for both physical activity and fitness? (yes/no).

Item 13. We will also be working with an honours student to conduct a feasibility study in regards to capturing ACT (and if consensus if reached, PA and fitness) data on the ACFDR. Please let us know in the text box below if you are interested in being on a working group for the feasibility study, or if you are interested in your site being involved in the feasibility study.
